# Enhancing motor learning of young soccer players through preventing an internal focus of attention: The effect of shoes colour

**DOI:** 10.1371/journal.pone.0200689

**Published:** 2018-08-15

**Authors:** Andrea De Giorgio, Maha Sellami, Goran Kuvacic, Gavin Lawrence, Johnny Padulo, Marco Mingardi, Luigi Mainolfi

**Affiliations:** 1 Faculty of Psychology, eCampus University, Novedrate, Italy; 2 Faculty of Kinesiology, University of Split, Split, Croatia; 3 Institute for the Psychology of Elite Performance. School of Sport, Health, and Exercise Sciences, Bangor University, Bangor, United Kingdom; 4 Tunisian Research Laboratory Sports Performance Optimization National Center of Medicine and Science in Sport, Tunis, Tunisia; Universidade de Tras-os-Montes e Alto Douro, PORTUGAL

## Abstract

The purpose of this research was to assess how the motor learning skills in 7-years old soccer players can be improved by preventing an internal focus of attention via the use coloured shoes. We painted the classic black soccer shoes in six areas corresponding to six regions of the foot with which it is possible to interact with the ball. Thirty-four 7-years-old soccer players were randomized to two groups (Coloured n = 17 and Black, n = 17) to perform four basic football manoeuvres/tasks: reception (RECP), passing (PASS), ball management (MAGT), and shooting (SHOT). We found highly significant differences (*P*<0.001) in all four performance tests: mean(sd) RECP: 0.82(0.07) vs. 0.45(0.12); PASS: 0.85(0.07) vs. 0.47(0.09); MAGT: 0.91(0.09); SHOT: 1.00(1.00) vs. 0.44(0.16). Colored shoes appear to draw children’s attention away from body centered cues without explicit verbal communications. We propose that this cognitive adaptation enhanced the technical gesture by preventing the negative processes associated with action constraining when adopting an internal focus attention (perhaps by allowing the foot to adapt to surfaces and movements more naturally than conditions that promote a focus on the body movement). Consequently, this type of coloured footwear could be used during childhood to allow children to enhance the performance of basic football exercises through preventing action constraining and promoting intuitive (non-body centered) action knowledge.

## Introduction

Soccer is a team sport where players are confronted with complex movements such as running with rapid changes of direction, with or without ball; and various kicking actions in specific and rapidly changing planes [1,2]. In fact, tactical skills are highly dependent on the interaction of the player with the surrounding information (constantly picking information’s from the ball, teammates and opponents), and his ability to anticipate future events is an integral part of skilled performance. As such a number of essential skills are needed to play soccer, such as physical characteristics, physiological demands, running economy, and cognitive abilities (tactical/strategic; perceptual/decision-making) that control and re-arrange tasks from the nervous system [3]. According to Gréhaigne and Godbout [4] strategic skills refer to elements of game play discussed in advance of the game which are designed to aid the team in organizing itself (e.g., understanding set plays, team composition) and the performers in understanding the roles of different positions. Tactical skills are player centred decisions made during the game in face the of dynamic changes (e.g., effective position shifts in response to an opponent’s action). Accordingly, Casanova et al., [5] and Grehaigne and Godbout [4] propose that tactical knowledge is essentially knowledge in action because tactical skills and performance are strongly linked. Thus, the development of tactical skills (effective individually based decision making) is an complex interaction between; the players knowledge of action rules i.e., important conditions and elements required for efficient action (e.g., if I want to ‘keep the ball’ I should protect the ball using my body); the rules for managing play organization i.e., principles that facilitate elaboration of a strategy (e.g., creating imbalance in ones favour), and perceptual and sensory-motor skills (i.e., the movement skills and the capabilities of players to recognize important perceptual information and act upon it). These perceptual skills consist of recognition, anticipatory cue extraction and use, visual search behavior [6] and acoustic detection [5].

In addition, perceptual skills including advanced visual learning capacity, visual recognition, visual search behavior, and acoustic learning [5,7] represent the “game intelligence” capacity as described by Stratton [7]. Visual, acoustic and temporal aspects are important to activate the focus of attention (FOA) of the athlete. An athlete’s FOA depends on external and internal factors that could alter learning and performance [8]. According to Wulf and colleagues [9], an external FOA encourages the athlete to focus on the effects of their movement on the environment, while an internal FOA encourages the athlete to think about his or her own body movement.

In this context, numerous studies have investigated the effect of different focus of attention on performance. Here, research has demonstrated that an external FOA enhances the motor learning of children and young performers compared to an internal FOA [9–16]. For example, Wulf and colleagues [9] investigated the learning effect of an external FOA in individuals with no experience in golf practice. Specifically, the authors asked participants to focus on either the arm swing (internal focus) or on the club swing (external focus) during a pitch shot in Golf. Results demonstrated that the group who received instruction to focus on the club swing performance significantly better than the group focusing on the arm swing. This pattern of results helps confirm the benefit from using an external FOA to enhance performance in individuals with no task experience (i.e., beginners).

Zachry et al., [17] also observed that participants who performed basketball free throws with instructions to focus on the basket (external FOA) had greater shot accuracy compared to participants who focused on their wrist motion (internal FOA). Furthermore, electromyography (EMG) data of the biceps and triceps muscle revealed reduced activity (more efficiency) in the external FOA group compared to the internal FOA group. Hence, it appears that an external FOA reduces “mechanical noise” and thus enhances movement economy compared to conditions that promote an internal FOA.

The benefits of adopting an external FOA over that of adopting an internal FOA has been proposed within the Constrained Action Hypothesis (CAH) [18]. Broadly speaking, two mechanisms are at play when attributing the benefits of an external over an internal FOA. 1) an internal FOA acts to constrain the normal automatic processes of movement and 2) an external FOA serves to enhance the congruity between action planning and movement outcome and thus increases the action-effect principle [19]. Evidence for such effects can be seen in the research of [20]. Here, baseball players who focused on an external irrelevant feature (i.e., participants who were prevented from focusing on the movements of the body but *not* explicitly told to focus on the effect of their movement) performed better than those required to focus internally, but not as well as those required to focus on the action of their movement. Thus, it appears that action performance can be enhanced by simply preventing an internal FOA, but enhanced further if that prevention also includes a strategy that focus attention of the effect of the action. In most previous studies, the strategy used to manipulate the external focus is done so through the use of explicit verbal instructions [18,21–23] or visual cues [24,25]. According to Gottlieb [26], attention to visual information searching is an important component of the cognitive system. During exercise, the motor system organizes itself towards the optimal movement pattern after receiving visual information. Since this process has been demonstrated to be easier for an expert or high level athlete compared to a beginner [24], it appears important to give clear and easy instructions to novice players [24] if performance benefits are the desired goal. For example, when a target is defined by a specific feature (i.e. form or colour) a young player and/or novice tend to respond quickly and more accurately regardless to the task [27]. In this context, Gibson [28], described the affordance concept as the process of constant characteristics of specific objects (i.e. form, size, color, texture, composition, motion, animation, and position relative to other objects) being directly perceived by the observer resulting in the presentation of the objects affordances. In other words, “Affordance” theory [28] explains the ability of the motor system to guide behavior or actions via interactions with the environment which results in directly perceiving what the environment offers in terms of potential and possible “action”. This theory describes a strong interaction between individual’s action and environment characteristic. However, the selection of affordances seems to face the problem of acquisition and development of actions in children, since maturity is a crucial element in the “perception-action” cycle [29,30] and affordances are partly determined by the performers action abilities [31]. Thus, if an external FOA enhances the action capabilities of a learner by preventing the proposed action constraining effects [18] of adopting an internal FOA, it is feasible that relatively more affordances are perceived under conditions of an external FOA.

Because children and novices are often in the cognitive stage of learning [32] their ability to pay attention to coaches instructions is limited relative to more intermediate and advanced performers. This is said to be because in early stages of learning performers are still trying to work out ‘what’ to do and ‘what’ is required of them and their motor system which consumes considerable attentional resources (see [32,33]) As such, it is important to develop new strategies or interventions to improve attention in this population. As such, improving attention through the development of the child’s visual search and target recognition strategies could be beneficial to the learning process in general. For example, recent literature using coloured targets such as colourful sport clothes and footwear have shown the importance of colour in enhancing mood, emotions, and even behavior. In fact, colour has been found to enhance individual’s arousal [34]. Wright [35] explained the effect of the electro-magnetic radiation of light on mood and behavior. He suggested that warm colour such as yellow, blue, green or red have a strong impact on the nervous systems response as they have the potential to stimulate the “hyperarousal” process (activation of stress hormones). For example, the uniform colour of goalkeepers (red vs. white) could influence the number of penalties scored [36]. In fact, penalty takers wearing red were perceived to possess positive characteristics to a greater extent than those wearing white. Moreover, goalkeepers reported higher expectancies of saving penalties from penalty takers wearing white uniforms than any other colour combination. These previous research articles investigating colour were focused on clothing colours while the colour of the shoe was not given consideration. There is one exception whereby Lam et al., [37] demonstrated that anaerobic performances (e.g., jump flight time) are enhanced in individuals wearing *warm* coloured shoes. In the game of soccer, the shoes (weight, form) are important for running and consequently playing ability [38]. As such they may well be perceived by the wearer as important for locomotor or action ability and thus partly determine the perceived affordances of the environment [31]. Furthermore, since strategies that prevent performers from focusing on their body actions result in an increase in action ability [20,21], it is possibility that using coloured shoes to prevent an Internal FOA may lead to increases in performance. Which in turn lead to increases in action ability and thus greater affordances compared to situations that promote an internal FOA. Thus, using strategically placed patterns of colour on youth soccer players shoes may be an effective and simple intervention to enhance soccer performance.

Consequently, the aim of the current study was to investigate if the use of coloured footwear prevents children from using an internal FOA (and thus constraining their actions) resulting in an increase in how they perform a series of basic football exercises.

## Materials and methods

### Participants

Thirty-four 7-years-old soccer players (height: 120.82± 3.50cm; body mass: 24.23 ± 1.87 kg) coming from the same football school, participated to this study. The Scientific committee of University of Split approved the entire study design which has been conducted according to the principles expressed in the Declaration of Helsinki. Moreover, parents of all children in this manuscript have given written informed consent (as outlined in PLOS consent form) to publish these case details. Eligible participants were subsequently randomized in experimental group (Coloured group; COLOUR, n = 17) or control group (Black group; BLACK, n = 17).

The control group wears the standard shoes which painted with black. Training status was assessed for all participants using validated Baecke questionnaire to quantify levels of activity [39]. All subjects were moderately trained and performed a one-year preparation in the local club. In addition, to identify those with a medical contraindication (exclusion criteria) to performing tests, participants completed medical history, and dietary, questionnaires. The inclusion criteria included: (i) at least one year of training experience (soccer only), (ii) having a valid sport medical certification including a standard ophthalmic exam (Visual acuity, refraction, and colour vision tests), and (iii) being healthy (i.e. absence of cardiovascular disease, no history of chronic disease, illness, surgeries, hospitalizations, and musculoskeletal or joint injuries).

### Testing procedures

**Fig 1:**
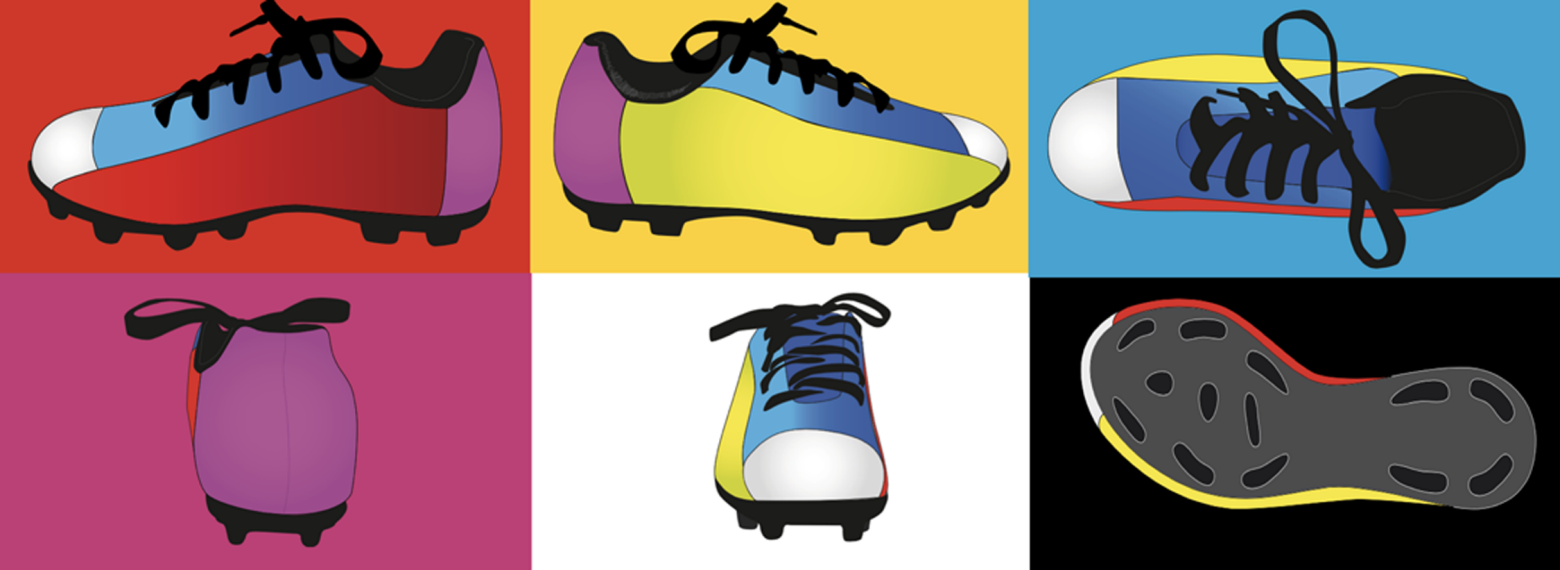
The image represents the exact subdivisions of the shoe. Red corresponds to the inside of the foot; yellow to the outside of the foot; blue to the neck of the foot; magenta to the heel; white to the tip of the foot; black to the sole.

For group design, the COLOUR wore a coloured shoe (Fig 1) starting from a classic black shoe. The shoe was divided into six squares / anatomical areas: toe tip (white); inner foot (red); outer foot (yellow); heel (magenta); sole (black); foot neck (blue). The choice of specific colour was based on scientific rational from Dzulkifli & Mustafar’s review [40]. In fact, the use of “Red”, “Yellow”, and “Magenta” (warm colour) was designed to increase attention of the young soccer [40]. In addition, the use of “white” colour on more dark background such as “Green”, “Blue”, or “Black” is more efficient for memory retention and attention [40]. Another factor that has been taken into account is the combination between different warm colour which appears important for memory performance and learning process [40].

For performances evaluation, the tests were held in the second part of the 2015/2016 season on four different periods for each test. All tests were performed on a regular soccer pitch with artificial grass in the morning (9–11 a.m.) to avoid any influence of circadian variation. Weather conditions were measured continuously during the tests: the temperature was situated between 20°C- 28°C, humidity: 22%-40%, wind: 1km/h - 2km/h, and the weather was clear during all the testing period.

The participant attended a familiarization session one week before the actual tests were performed. During this session, all participants had to perform all tests following same conditions (weather, clothes and monitor) and instructions. The same devices were also used to record data including stopwatch (chronometer). Stopwatches were calibrated before the start of trials (calibration performed against another stopwatch of a second investigator). All recorded data from familiarization session were only used to study the feasibility and to learn about level and score before the start [41].

All sessions (familiarization and tests) were carried out in the same conditions and coached by the principal instructor who gave instructions, explained the procedures and monitored tests, a second expert recorded scores and a third one controlled the testing procedure conditions (i.e. materials, population requirements etc).

During session, all participants wore “White” colour soccer uniforms. Each group was subjected to four different performance tests after a standard warm-up (10’ of jogging and 3’ of dynamic stretching): reception (RECP), passing (PASS), management (MAGT) and shooting (SHOT).

For each test performance “1” point was attributed if the performance was carried out properly and “0” when improperly performed. For the COLOUR, the performance was considered wrong when the ball was hit with the wrong colour. For the BLACK, the performance was considered wrong when the child hit the ball not following the coach’s instructions. There was an exception in SHOT, in which “1” point was attributed when both conditions were satisfied: when the ball was hit with the correct part of the shoe (according to colour or coach’s instructions) and the ball passed between cones (further information in paragraph 2.2.4).

#### Reception test–RECP (Fig 2A)

(*You can see a video example here* *https://figshare.com/s/3478dddbbebbc0e7aaba*)

**Fig 2:**
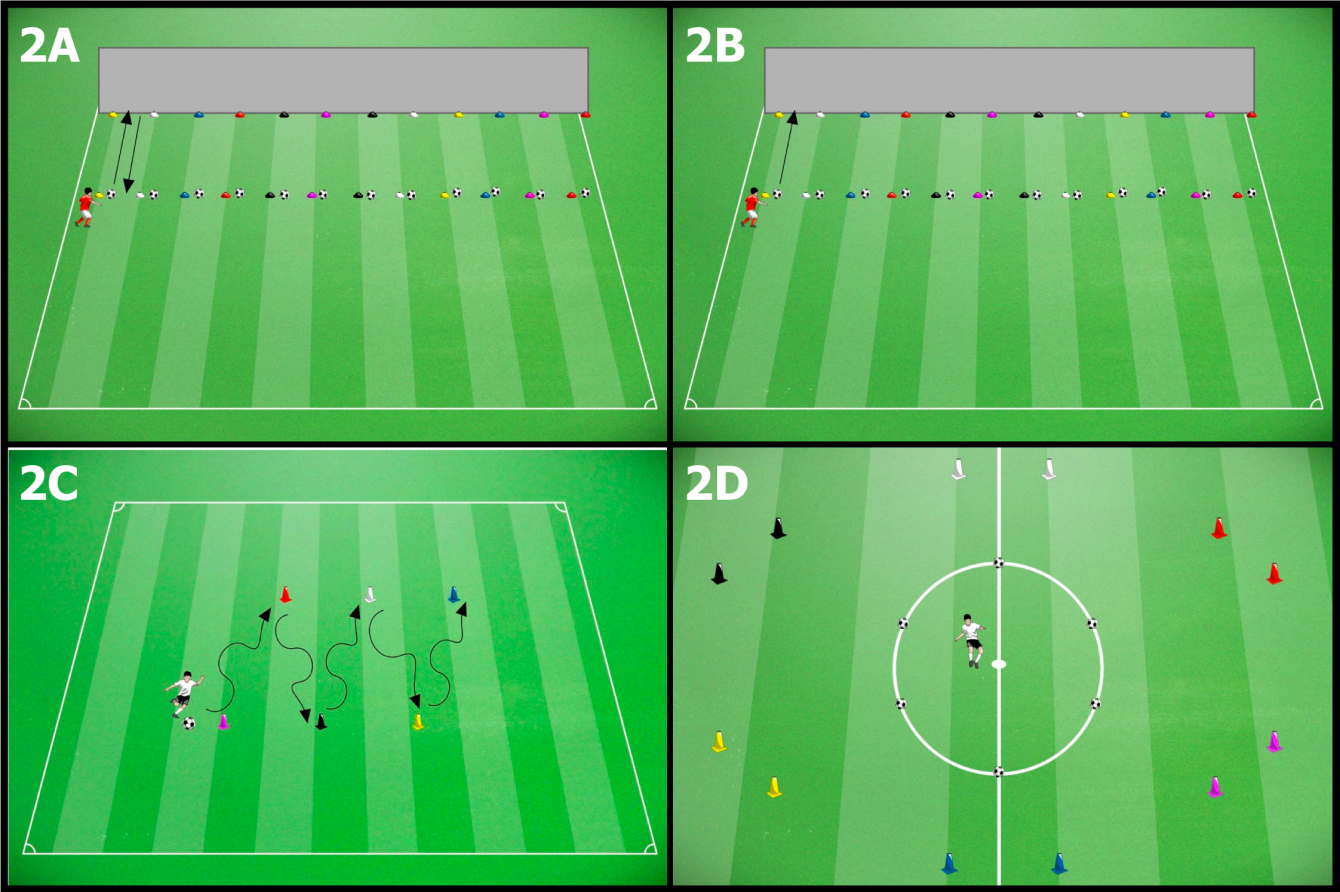
Receiving (A); passing (B); management (C); and shooting (D) tasks.

**Material**: The test area was set at 25×42 meters; 12 footballs measure 4 in white colour, 12 boundary markers (2 yellow, 2 white, 2 magenta, 2 red, 2 blue and 2 black).

**Method**: The different coloured boundary markers were placed so that the same colour did not repeat consecutively. The markers were located at a distance of 1.50 m from the wall and the next marker. The participant hit the ball freely towards the wall (i.e., without paying attention to the colour) to receive it back. Twelve trials were then performed.

COLOUR: the participant hit the ball and directed it to the wall freely (i.e., without paying attention to the colour) but received it with the part of the shoe corresponding to the colour of the boundary marker. In this case, the coach explained the children, before the whole trial, to hit the ball by associating the colour of the shoe to the colour of the boundary marker.

BLACK: the participant hit the ball and directed it towards the wall freely and received it with the anatomical part indicated by the trainer (Table 1). For each individual reception, the coach changed the indication of the anatomical part of the foot used to play the ball.

With regard to instructions, BLACK group received each coach’s instructions just before each boundary marker as summarized in **Table 1**. In order to allow better reaction time, the following instructions or auditory stimulus which lasted < 1, 00 second, were given always by the same expert to maintain sound’s amplitude and tone. It is important to note that all instructions have been also explained and repeated during familiarization session (before start of trials). Therefore, we ensure that all instructions are well understood, facilitate listening during motion and reduce acoustic noises effects.

**Table 1:** Coach’s instructions for each test for BLACK group.

	Inside of the foot	Outside of the foot	Tip of the foot
**RECP**	*Receive the ball with the inside*	*Receive the ball with the outside*	*Receive the ball with the tip*
**PASS**	*Hit with the inside*	*Hit with the outside*	*Hit with the tip*
**MAGT**	*Touch with the inside*	*Touch with the outside*	*Touch with the tip*
**SHOT**	*Kick with the inside*	*Kick with the outside*	*Kick with the tip*
	**Instep of the foot**	**Sole of the foot**	**Heel of the foot**
**RECP**	*Receive the ball with the instep*	*Receive the ball with the sole*	*Receive the ball with the heel*
**PASS**	*Hit with the instep*	*Hit with the sole*	*Hit with the heel*
**MAGT**	*Touch with the instep*	*Touch with the sole*	*Touch with the heel*
**SHOT**	*Kick with the instep*	*Kick with the sole*	*Kick with the heel*

#### Passing test–PASS (Fig 2B)

(*You can see a video example here*: *https://figshare.com/s/095225ffa63fdeda17f6*)

**Material**: The test area was set at 25×42 meters, 12 footballs measure 4 in white colour, 12 boundary markers (2 yellow, 2 white, 2 magenta, 2 red, 2 blue and 2 black).

**Method**: the boundary markers of different colours were placed so that the same colour did not repeat consecutively. The markers were located at a distance of 1.50 m from the wall and the next marker. The participant hit the ball and directed it to the wall (i.e., without paying attention to the colour). Twelve trials were then performed.

COLOUR: the participant hit the ball and directed it to the wall with the anatomical part of the foot corresponding to the colour of the boundary marker. Before the whole trial, the coach gives instruction to hit the ball by associating the colour of the shoe to the colour of the boundary marker.

BLACK: the participant hit the ball and directed it towards the wall with the anatomical part indicated by the trainer (Table 1). For each individual reception, the coach changed the indication of the anatomical part of the foot used to play the ball.

#### Management test–MAGT (Fig 2C)

(*You can see a video example here*: *https://figshare.com/s/664d2068aaca84dd7f85*)

**Material**: The test area was set at 25×42 meters, 12 balls measure 4 in white colour, 6 pairs of cones (1 yellow, 1 white, 1 magenta, 1 red, 1 blue and 1 black).

**Method**: Cones of different colour have been positioned so that the same colour did not repeat consecutively. The cones were positioned 6 m away from each other. The participant led the ball between a pair of cones and the other.

COLOUR: the participant led the ball with the foot part corresponding to the colour of the cones until the test was completed. Before the whole trial, the coach gives instruction to use the ball by associating the colour of the shoe with the colour of the cones.

BLACK: the participant led the ball with the anatomical part indicated by the trainer (Table 1). For each individual reception, the coach changed the indication of the anatomical part of the foot with which to play the ball.

#### Shooting test–SHOT (Fig 2D)

(*You can see a video example here*: *https://figshare.com/s/7317cb9e470a180e7ff8*)

**Material:** the test area was set at 25×42 meters, 12 balls measure 4 in white, 6 pairs of cones (2 yellow, 2 white, 2 magenta, 2 red, 2 blue and 2 black).

**Method**: the participant is placed in the center of a square (4x4 meters) defined by four orange boundary markers (i.e., colour not on the shoe). At 12 meters from the center of the square, there are 6 goals measuring 2 meters of the same colour corresponding to one of the 6 anatomical areas of the shoe. The ball is positioned halfway between the central square and the goal. The participant can kick any ball (i.e., he does not follow a timetable or counter clockwise) provided that after each single kick it returns to the central square.

COLOUR: the participant kicked the ball with the anatomical part of the foot corresponding to the colour of the goal; when he returned to the square, the child decided independently which ball to hit and towards which goal. In this case, the coach just explained the children, before the whole trial, to use the ball by associating the colour of the shoe with the colour of the cones.

BLACK: the participant kicked the ball with the anatomical part of the foot indicated by the coach (Table 1); when he returned to the square the coach indicated a new anatomical part. The child was free to hit the ball without following a clockwise or anti-clockwise direction.

### Statistical analyses

All the variables were tested for normality by the Kolmogorov Smirnov with Lilliefors correction. Assumptions of normality of distribution were violated for all performance variables (total score) in both groups. For performance indices over the four variables considered (reception, passing, management, shooting), data were analyzed using non-parametric Mann Whitney U test. Additionally, raw and standardized (Cohen’s d) differences in mean with 90% confidence intervals were calculated for comparisons between groups. Threshold values of Cohen’s d were: trivial = < 0.20; small = 0.2–0.59; moderate = 0.60–1.19; large = 1.20–1.99; very large = > 2.0 [42]. To better understand the real differences, magnitude-based inferences were determined by quantifying the chances that true differences were greater than, similar to, or smaller than the smallest worthwhile difference (SWD—0.2 multiplied by the between-participants deviation) and interpreted qualitatively as: almost certainly not = < 0.5%; very unlikely = 0.5–5%; unlikely = 5–25%; possible = 25–75%; likely = 75–95%; very likely = 95–99.5%; almost certain = > 99.5% [43]. A *p* value of less than 0.05 was regarded as a statistically significant difference. Statistical analyses were carried out using STATISTICA software version 13.0 (Dell Inc., Round Rock, TX USA) and spreadsheets [44,45] in Microsoft Excel (Microsoft Corporation; Redmond, WA, USA).

## Results

During the experimental period, there were no significant differences (*p*>0.05) in anthropometric measurement, however, several significant differences were observed in performances results. Differences between experimental (COLOUR) and control group (BLACK) in scores and time for the four performance tests (reception, passing, management and shooting) are presented in Table 2.

**Table 2:** Differences in scores and time for performance tests (reception, passing, management and shooting).

Variables			Difference between groups^a^				DM^b^ (with 90% CI)		MBI^c^	
			COLOUR (n = 17)		BLACK (n = 17)		Raw data	Cohen’s *d*	%chance	Interpretation
*score*	RECP	Mean (SD)	9.82	(0.88)	5.41	(1.46)**	4.41(3.71 to 5.11)	3.66(2.66 to 4.48)	100/0/0	*Most likely beneficial*
		Median (range)	10	(9–11)	5	(2–8)				
	PASS	Mean (SD)	10.24	(0.83)	5.65	(1.06)**	4.59(4.04 to 5.14)	4.82(3.61 to 5.80)	100/0/0	*Most likely beneficial*
		Median (range)	10	(9–12)	6	(4–7)				
	MAGT	Mean (SD)	5.47	(0.51)	2.65	(0.93)**	2.82(2.39 to 3.26)	3.76(2.75 to 4.60)	100/0/0	*Most likely beneficial*
		Median (range)	5	(5–6)	3	(1–4)				
	SHOT	Mean (SD)	6.00	/	5.18	(0.53)**	0.82(0.61 to 1.04)	2.19(1.43 to 2.84)	100/0/0	*Most likely beneficial*
		Median (range)	6	(6–6)	5	(4–6)				
*time*	RECP	Mean (SD)	75.41	(6.82)	76.06	(13.95)	-0.65(-7.03 to 5.73)	-0.06(-0.62 to 0.51)	23.7/40.9/35.3	*Unclear*
		Median (range)	76.00	(62–88)	77.00	(46–96)				
	PASS	Mean (SD)	72.53	(8.39)	80.47	(11.64)*	-7.94(-13.84 to -2.05)	-0.78(-1.35 to -0.18)	0.4/4.5/95.1	*Very likely beneficial*
		Median (range)	72.00	(61–91)	79.00	(62–100)				
	MAGT	Mean (SD)	74.00	(8.82)	81.47	(12.26)	-7.47(-13.68 to -1.26)	-0.70(-1.26 to -0.10)	0.7/7.0/92.3	*Likely beneficial*
		Median (range)	72.00	(62–94)	82.00	(64–100)				
	SHOT	Mean (SD)	21.18	(5.20)	24.47	(6.45)	-3.29(-6.70 to 0.11)	-0.56(-1.12 to 0.03)	1.7/13.2/85.1	*Likely beneficial*
		Median (range)	21.00	(13–33)	23.00	(16–37)				

Data presented as mean (SD) and median (range). RECP–receiving test. PASS–passing test. MAGT–management test. SHOT–shooting test. COLOUR–experimental group. BLACK–control group, CI–confidence intervals, %chance–higher/similar/lower

*p<0.05,

**p<0.001,

^a^Mann-Whitney U test,

^b^differences in mean,

^c^magnitude-based inference.

For scores, highly significant differences were observed between COLOUR and BLACK (*p*<0.001) with differences in mean (with 90%CI; 4.41(3.71 to 5.11, 4.59(4.04 to 5.14), 2.82(2.39 to 3.26), 0.82(0.61 to 1.04)) demonstrating very large effect size (with 90%CI; 3.66(2.66 to 4.48), 4.82(3.61 to 5.80), 3.76(2.75 to 4.60), 2.19(1.43 to 2.84)) in RECP, PASS, MAGT and SHOT, respectively.

Interestingly, in SHOT performances, COLOUR performed all the kicks properly compared to BLACK (mean (SD); 6.00(/) vs. 5.18(0.63).

For performance-time (or time to achieve exercise), statistically significant difference was observed in PASS (U = 84.50, *p*<0.05). Also, we found differences in mean (-7.47(-13.68 to 1.26), 3.29(-6.70 to 0.11)) demonstrating moderate effects size (0.70(-1.26 to -0.10), -0.56(-1.12 to 0.03)) in MAGT and SHOT, respectively. Smallest observed difference between COLOUR and BLACK was in RECP (-0.06(-0.62 to 0.51) and -0,65(-7.03 to 5.73)).

With magnitude-based inherence approach, comparison between groups produced *most likely beneficial* differences in all variables for score with smallest worthwhile differences (SWD) ranging from 0.053 to 0.234. For performance-time, these differences were *unclear* (RECP), very likely beneficial (PASS), and likely beneficial (MAGT and SHOT), where SWD ranged from 1.165 to 2.019

## Discussion

The purpose of this research was to investigate whether using coloured shoes, acts as an external focus of attention by preventing the constraining processes associated with adopting an internal focus of attention, resulting in improvements in motor learning skills (i.e. shooting, pass, reception, and management) in 7-years old soccer. This intervention intended to help recognize all parts of the foot intuitively and to decrease the time of execution in basic football exercises in young soccer. Results indicated that players wearing coloured shoes performed significantly better (i.e. reception, shooting, passing and ball management tasks) than their counterparts in the control group.

Similar results were also reported by numerous studies. In fact, it has been found that golf performance was increased when focus was directed on either the swing of the club, the clubface, or the intended ball trajectory rather than on the wrist movement [46] or arms [23]. In basketball, higher scores in free throw performance were registered in the group who focused attention on ball trajectory relative to basket compared to group who focused attention on the gesture (i.e., upper leg movement and, in particular, of the wrist [47]). In football, higher hitting performance was recorded when subject’s attention is directed to the part of the ball required to strike (i.e., external stimuli) rather than the part of the foot they would have to use to make contact with the ball (i.e., internal [17]).

Many researchers have confirmed the strong impact of the external FOA on the effectiveness and accuracy of motor performance [12,48] and on the speed of the learning process [12]. In the current study, when the performer was required to direct their attention to the targeted colour of the shoe, they are prevented from directing their attention to the movements themselves (internal feedback) which had greater impact on decreasing the task execution time. Wulf, and Colleagues [10,18,23] advocate that two mechanisms are at play when attributing the benefits of an external over an internal FOA. 1) an internal FOA acts to constrain the normal automatic processes of movement and 2) an external FOA serves to enhance the congruity between action planning and movement outcome and thus increases the action-effect principle [19]. As hypothesized, it appears that the colour condition in the current experiment acted to prevent the first process from happening, resulting in increased performance relative to the internal FOA condition. That is, the colour intervention prevented or reduced the constraining effects associated with adopting an explicit internal (body centered) focus of attention (internal FOA). In line with this suggestion, previous literature [20] indicates that adopting an environmental focus (i.e., a focus that is outside of the body, but not explicitly centered toward the effects of a movement e.g., focusing on an auditory tone, focusing on footwear colour and its contact with the ball) results in greater performance compared to adopting an internal FOA. In a similar vein, Lawrence et al., [22] reported that under both internal and external FOA conditions participants create a comparable number of explicit cues/rules regarding the movement technique. The performance benefits of adopting an external FOA in Lawrence et al., [22] were attributed to these explicit cues/rules being environmental focused and not body centered (thus preventing the constraining processes proposed within the CAH) in the external FOA condition. Whilst the current experiment did not measure the number or type of explicitly generated cues/rules, it is possible that the benefits of the Colour condition over that of the IFOA condition are due, in part, to this effect. Whilst the benefits of an EFOA that is directed toward a movement effect (e.g., the manipulations of Wulf and Colleagues [9]) is proposed to be due to both preventing action constraining and enhancing action planning via processes within the action-effect principle (see [21]), our colour manipulation did not directly asks participants to focus on a movement effect. However, we still observed increased performance relative to the internal FOA group. As such, the performance benefits of the Colour condition over that of the IFOA condition are likely due to the condition *only* preventing the constraining processes proposed to occur when one adopts an IFOA rather than the additional action-effect principles associated with adopting a focus that directs attention to the effects of one’s movement.

The use of color as a target for attraction appears to have excellent impact on learning process and memory [49,50]. As we know, cognitive skills depend on perception, attention, memory and the way of thinking of an individual. Therefore, to facilitate this process, there must be intervention/method to facilitate “perception” first through exposing eye to attractive information (i.e. light). In fact, when persons are exposed to different colors and pictures, they were attracted “spontaneously” to warm colours (red, blue, green, yellow [51,52].

Furthermore, this is said that children and novices are in early stages of learning and are thus still trying to work out ‘what’ to do and ‘what’ is required of them and their motor system which consumes considerable attentional resources (see [32,33]). As such, it is important to develop new strategies to improve attention and memory in this population. There is evidence that visual memory and recognition are more robust than auditory memory [53]. The rationale being that the auditory received verbal instruction, has to be transformed into wave information, travel across the auditory system before being transformed into electric impulse to be recognized by the brain. Hence, to recognize the part of the foot to use during hitting ball, the verbal instruction of the coach must pass through a complex system. However, when the task is combined with visual searching for example (choosing the part of the foot to hit the ball, how to hit the ball and direction of the goal), the perception system will focus on the most vital task and then the information that should be treated first will be chosen according to level of motivation, attention and concentration. This suggests that visual information will be process/treated first. Since this strategy is more useful for beginner due the reduced attention compared with a high level athlete [18,54,55], we propose that the colour condition aided in performance strategy similar to that proposed above which impacted towards our observed performance gains. Furthermore, in relation to Fajen and Matthis [31] action-scaled affordances approach, we speculate that this increase in performance and locomotor action, lead to the direct perception of a greater number of affordances compared to the internal FOA group. That is, if the number of possible actions (i.e., affordances) are partly determined by the motor capabilities of the performer, then those participants in the colour group should have been afforded more possible actions as a direct result of their improved performance.

Although the findings of this study make a unique contribution to the fields of exercise science and motor learning, it is worth mentioning that the current research used basic football tasks that are not representative of the dynamic and multifaceted game of soccer. Indeed, it was not the intension of the experiment that the research design promote external validity over internal validity; these two concepts are paradoxical in nature whereby increasing one results in a reduction in the other. However, the central point of the investigation (the manipulation of footwear colour as a way to prevent an internal FOA) was novel within the FOA scientific literature. As such, we deemed it essential to adopt an experimental design that allowed for as rigorous internal validity as possible whilst still adopting tasks that were relevant to the game of soccer; if not completely representative of the game itself. The rationale being that, in order to establish the hypothesized cause and effect of a coloured footwear intervention enhancing performance over that of an internal FOA we needed to ‘sacrifice’ external validity in favour of internal validity. As such, the results should be applied to soccer with caution until they are tested in an externally valid experimental design that uses tasks representative of the complete dynamic and multifaceted real-world game of soccer.

Apparently, these quick adjustments in performance (short-term changes) in our participants are due to enhancement of temporary storage of information. Since, colour is easier to understand than verbal communication, we assumed that this temporary storage of information would allow the information to be used for ongoing mental processes. As such, long-term studies should be addressed to assess how young players technical performance could be improved across a long-period. In addition, the use of the external focus of attention during game would become an essential element of training and can be simply included in different situations to enhance young soccer player performance.

On the other hand, current findings may provide the most informative approach given the need to study the physiological process that lie behind these improvements and if possible to adapt this strategy in young player regardless to their gender. In fact, it will inform policy and practical solutions for coaches during training sessions especially at the beginning of the season to facilitate motor learning in young players (≤14 years). In addition, this intervention maybe an easy digestible strategy for individuals with hearing impairment or might have trouble understanding verbal instructions and extend theory in health community as a motivational intervention.

## Conclusion

In summary, the current study has clearly demonstrated that coloured shoes have a positive effect on motor performance relative to conditions that promote an internal FOA. We suggest this occurs because of the interactive effects of the coloured shoe acting to prevent an internal (body centered) FOA, resulting in actions that are not constrained, and therefore providing the direct perception of a greater number of affordances in comparison to an explicit verbal instruction group that promotes the adoption of an internal FOA. Whilst these results are promising to the world of soccer as a simple coaching ‘tool’ designed to improve the performance of youth players, the current research used basic football tasks. As such, until the intervention tested in an externally valid experimental design that uses tasks representative of the complete dynamic and multifaceted real-world game of soccer, the findings should be applied to with caution.

## Supporting information

S1 Fig Colour subdivisions of the shoe. The image represents the exact subdivisions of the shoe. Red corresponds to the inside of the foot; yellow to the outside of the foot; blue to the neck of the foot; magenta to the heel; white to the tip of the foot; black to the sole.
(TIFF)

S2 Fig Receiving (A); passing (B); management (C); and shooting (D) tasks.
(TIFF)

S1 Table Coach’s instructions for each test for BLACK group. (DOCX)

S2 Table Differences in scores and time for performance tests (reception, passing, management and shooting). Data presented as mean (SD) and median (range). RECP–receiving test. PASS–passing test. MAGT–management test. SHOT–shooting test. COLOUR–experimental group. BLACK–control group, CI–confidence intervals, %chance–higher/similar/lower *p<0.05, **p<0.001, ^a^Mann-Whitney U test, ^b^differences in mean, ^c^magnitude-based inference.
(DOCX)
