# Supplementary figures and images for: Enhancing motor learning of young soccer players through preventing an internal focus of attention: The effect of shoes colour
Source: PLoS One. 2018 Aug 15;13(8):e0200689. doi: 10.1371/journal.pone.0200689 (PMC6093605; doi:10.1371/journal.pone.0200689)

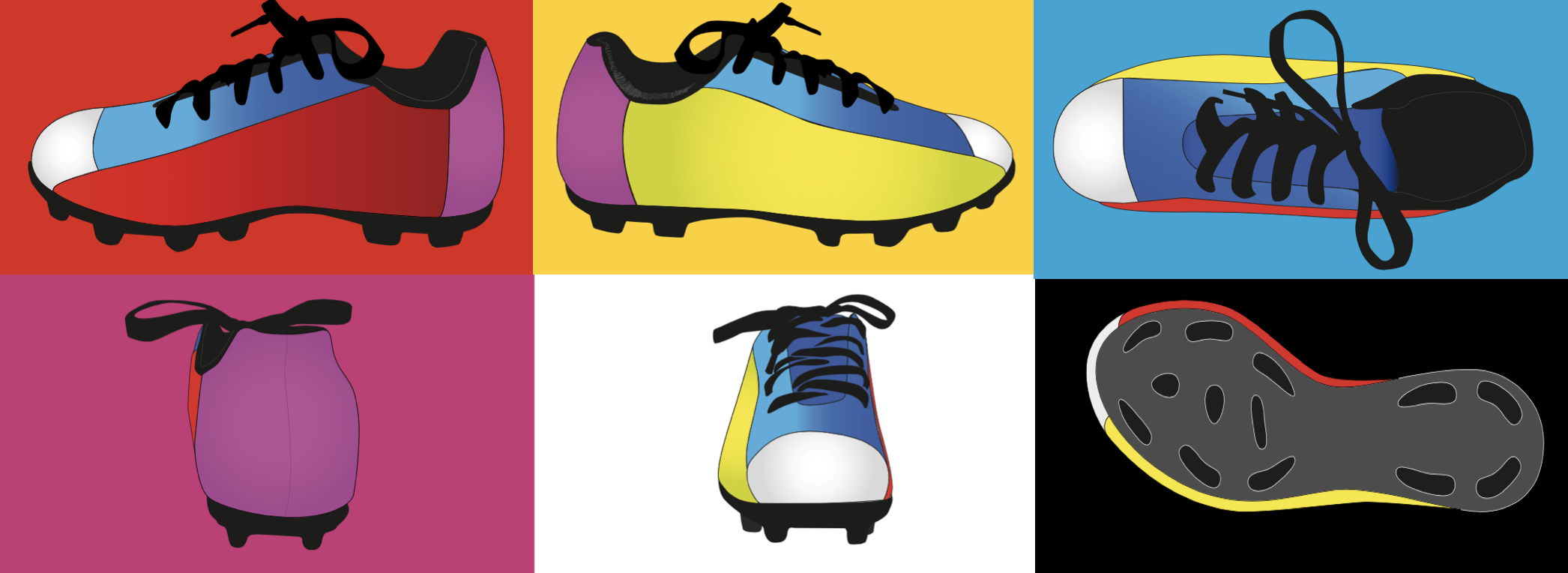

Supplement: S1 Fig — The image represents the exact subdivisions of the shoe. Red corresponds to the inside of the foot; yellow to the outside of the foot; blue to the neck of the foot; magenta to the heel; white to the tip of the foot; black to the sole. (TIFF) [file pone.0200689.s001.tiff]

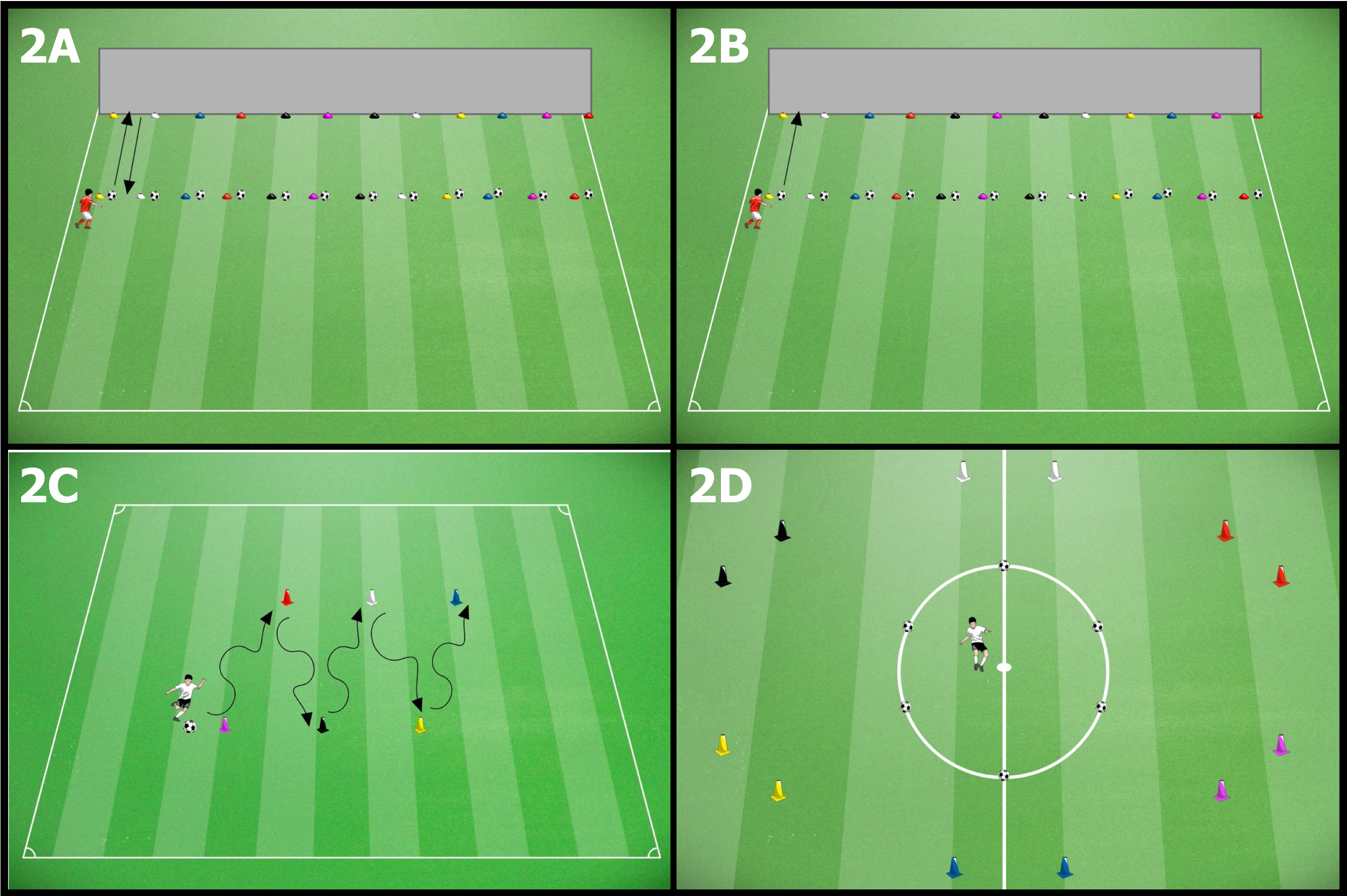

Supplement: S2 Fig — Receiving (A); passing (B); management (C); and shooting (D) tasks. (TIFF) [file pone.0200689.s002.tiff]
