# Supplementary material for: Enhancing motor learning of young soccer players through preventing an internal focus of attention: The effect of shoes colour
Source: PLoS One. 2018 Aug 15;13(8):e0200689. doi: 10.1371/journal.pone.0200689 (PMC6093605; doi:10.1371/journal.pone.0200689)
Supplement: S1 Table — (DOCX) [file pone.0200689.s003.docx]

**Table 1.** Coach’s instructions for each test for BLACK group

|  | **Inside of the foot** | **Outside of the foot** | **Tip of the foot** |
| --- | --- | --- | --- |
| **RECP** | *Receive the ball with the inside* | *Receive the ball with the outside* | *Receive the ball with the tip* |
| **PASS** | *Hit with the inside* | *Hit with the outside* | *Hit with the tip* |
| **MAGT** | *Touch with the inside* | *Touch with the outside* | *Touch with the tip* |
| **SHOT** | *Kick with the inside* | *Kick with the outside* | *Kick with the tip* |
|  | **Instep of the foot** | **Sole of the foot** | **Heel of the foot** |
| **RECP** | *Receive the ball with the instep* | *Receive the ball with the sole* | *Receive the bal lwith the heel* |
| **PASS** | *Hit with the instep* | *Hit with the sole* | *Hit with the heel* |
| **MAGT** | *Touch with the instep* | *Touch with the sole* | *Touch with the heel* |
| **SHOT** | *Kick with the instep* | *Kick with the sole* | *Kick with the heel* |
