# Supplementary material for: Enhancing motor learning of young soccer players through preventing an internal focus of attention: The effect of shoes colour
Source: PLoS One. 2018 Aug 15;13(8):e0200689. doi: 10.1371/journal.pone.0200689 (PMC6093605; doi:10.1371/journal.pone.0200689)
Supplement: S2 Table — Data presented as mean (SD) and median (range). RECP–receiving test. PASS–passing test. MAGT–management test. SHOT–shooting test. COLOUR–experimental group. BLACK–control group, CI–confidence intervals, %chance–higher/similar/lower *p<0.05, **p<0.001, aMann-Whitney U test, bdifferences in mean, cmagnitude-based inference. (DOCX) [file pone.0200689.s004.docx]

**Table 2.** Differences in scores and time for performance tests (reception, passing, management and shooting) .

| **Variables** | |  | **Difference between groups^a^** | | | | **DM^b^ (with 90% CI)** | | **MBI^c^** | |
| --- | --- | --- | --- | --- | --- | --- | --- | --- | --- | --- |
|  |  |  | COLOUR (n=17) | | BLACK (n=17) | | Raw data | Cohen’s *d* | %chance | Interpretation |
| *score* | RECP | Mean (SD) | 9.82 | (0.88) | 5.41 | (1.46)****** | 4.41(3.71 to 5.11) | 3.66(2.66 to 4.48) | 100/0/0 | *Most likely beneficial* |
|  |  | Median (range) | 10 | (9-11) | 5 | (2-8) |  |  |  |  |
|  | PASS | Mean (SD) | 10.24 | (0.83) | 5.65 | (1.06)****** | 4.59(4.04 to 5.14) | 4.82(3.61 to 5.80) | 100/0/0 | *Most likely beneficial* |
|  |  | Median (range) | 10 | (9-12) | 6 | (4-7) |  |  |  |  |
|  | MAGT | Mean (SD) | 5.47 | (0.51) | 2.65 | (0.93)****** | 2.82(2.39 to 3.26) | 3.76(2.75 to 4.60) | 100/0/0 | *Most likely beneficial* |
|  |  | Median (range) | 5 | (5-6) | 3 | (1-4) |  |  |  |  |
|  | SHOT | Mean(SD) | 6.00 | / | 5.18 | (0.53)****** | 0.82(0.61 to 1.04) | 2.19(1.43 to 2.84) | 100/0/0 | *Most likely beneficial* |
|  |  | Median (range) | 6 | (6-6) | 5 | (4-6) |  |  |  |  |
| *time* | RECP | Mean (SD) | 75.41 | (6.82) | 76.06 | (13.95) | -0.65(-7.03 to 5.73) | -0.06(-0.62 to 0.51) | 23.7/40.9/35.3 | *Unclear* |
|  |  | Median (range) | 76.00 | (62-88) | 77.00 | (46-96) |  |  |  |  |
|  | PASS | Mean (SD) | 72.53 | (8.39) | 80.47 | (11.64)***** | -7.94(-13.84 to -2.05) | -0.78(-1.35 to -0.18) | 0.4/4.5/95.1 | *Very likely beneficial* |
|  |  | Median (range) | 72.00 | (61-91) | 79.00 | (62-100) |  |  |  |  |
|  | MAGT | Mean (SD) | 74.00 | (8.82) | 81.47 | (12.26) | -7.47(-13.68 to -1.26) | -0.70(-1.26 to -0.10) | 0.7/7.0/92.3 | *Likely beneficial* |
|  |  | Median (range) | 72.00 | (62-94) | 82.00 | (64-100) |  |  |  |  |
|  | SHOT | Mean(SD) | 21.18 | (5.20) | 24.47 | (6.45) | -3.29(-6.70 to 0.11) | -0.56(-1.12 to 0.03) | 1.7/13.2/85.1 | *Likely beneficial* |
|  |  | Median (range) | 21.00 | (13-33) | 23.00 | (16-37) |  |  |  |  |

Data presented as mean (SD) and median (range). RECP – receiving test. PASS – passing test. MAGT – management test. SHOT – shooting test. COLOUR – experimental group. BLACK – control group, CI – confidence intervals, %chance – higher/similar/lower **p*<0.05, ***p*<0.001, ^a^Mann-Whitney U test, ^b^differences in mean, ^c^magnitude-based inference.
